# Supplementary material for: Machine Learning and Antibiotic Management
Source: Antibiotics (Basel). 2022 Feb 24;11(3):304. doi: 10.3390/antibiotics11030304 (PMC8944459; doi:10.3390/antibiotics11030304)
Supplement: Supplementary file 1 [file antibiotics-11-00304-s001.zip › antibiotics-1579233-supplementary.pdf]

Machine Learning and Antibiotic Management

Supplementary Material

In the paper, we propose cluster analysis performed for normalized data (according to fuzzy methods as described in the text) using k-modes algorithm for categorical values. We decided to show the results obtained performing k-means clustering on the same datasets as comparison.

The number of clusters for every k-means analysis was calculated computing average Silhouette Coefficient (SC) width [1, 2] for each k in a starting range of 25 (random start for centroids coordinates and 300 repetitions each).

The SC represents how well each case (i.e., rows in monitoring or therapy dataset) lies within its cluster and how appropriate each case's assignment is within a specific cluster.

We used the Silhouette Visualizer from the Yellowbrick library for Python [3]. The Silhouette Visualizer displays the silhouette coefficient for each sample on a per-cluster basis, visually evaluating the density and separation between clusters. The score is calculated by averaging the silhouette coefficient for each sample, computed as the difference between the average intra-cluster distance and the mean nearest-cluster distance for each sample, normalized by the maximum value. This produces a score between -1 and +1, where scores near +1 indicate high separation and scores near -1 indicate that the samples may have been assigned to the wrong cluster.

We decided to use 4 (score 0.10) clusters in the therapy dataset and 7 clusters (score 0.28) in the monitoring set for k-means analysis.

In the paper we decided to use KModes Clustering Algorithm for Categorical data. We performed clustering on data converted to Fuzzy subset categorical value. We show plots of elbow method used to choose the number of clusters (in figures 3 and 4 cost is the sum of all the dissimilarities between the clusters: we selected the K where an elbow-like bend with a lesser cost value was observed) and present again tables of frequencies for fuzzy subsets distribution among the clusters in the two datasets (Monitoring and Therapy).

We propose the comparison of the two kinds of clustering as an indirect sign that our approach could lead, in a more detailed further analysis, to interesting results for clinicians. Using both methods (one more appropriate for categorical values like the fuzzy subsets, the other more robust and validated for continuous values), we can observe clustering (apparently similar) for the monitoring and therapy datasets.

Performing K-means cluster analysis, we observed that the distribution of the observations in the Therapy dataset showed a concentration of most cases in cluster 1 and in the Monitoring dataset in cluster 1, 2 and 6.

In the K-modes analysis most of the Therapy cases were in cluster 0 and in the Monitoring dataset in cluster 0, 1, 2.

Looking at the Fuzzy subsets (categories) frequency distribution among clusters in Therapy dataset either in k-means and in K-modes analysis, we can see that most observations in k-means cluster 1 are F1 and F2 (corresponding to "very low" - 39% - and "low" - 50% - categories). This is similar to our findings in K-mode analysis where for the Therapy dataset cluster 0 F1 ("very low") is 24% and F2 ("low") is 38%.

For K-means analysis: examining the frequency distribution of fuzzy subsets in each therapy cluster, in cluster 1 and 2 (T1 and T2 in tables and charts) more fuzzy subsets F1 (39% in T1; 29% in T2) and F2 (50% in T1 and 41% in T2) (representing "very low" and "low" duration of therapy) may be found, and in cluster 3 (T3) there are more of summed "high" and "very high" fuzzy subsets (F4 and F5 = 41%), meaning long and very long duration of therapy (but also 30% of F2="low" duration).

In cluster 1 (T1) there is the highest number of fuzzy subsets F1 (17%) and F2 (59%) ("very low" and "low" duration) considering the overall duration of (any) antimicrobial therapy.

Monitoring clusters 0 and 2 (M0 and M2 in tables and charts) have more fuzzy subsets F4 and F5 (35% in M0 and 40% in M2), while M4 and M6 have more F1 and F2 (M4 55%; M6 44%) than the others.

In our population using k-means clustering the maximum number of therapy clusters per patient ICU stay was 4 (i.e. there were patients that during the ICU stay changed therapy pattern and overall duration of therapy to a maximum of 4 different clusters). Patients with only one therapy cluster during ICU stay had therapy days mostly in cluster T1; patients with 4 clusters in the ICU stay had equal frequencies of the 4 therapy clusters.

For k-modes analysis: looking at the frequency distribution of fuzzy subsets in each therapy cluster, in cluster 1 and 2 (T1 and T2 in tables and charts) more fuzzy subsets F1 (11% in T1; 21% in T2) and F2 (49% in T1 and 47% in T2) (representing "very low" and "low" duration of therapy) may be found, and in cluster 3 (T3) there are more of summed "high" and "very high" fuzzy subsets (F4 and F5 = 36%), meaning long and very long duration of therapy (but also 34% of F2="low" duration).

Cluster T0 has a very low percentage of "long" and "very long" duration of therapy (F4 = 9%, F5 =7%).

Monitoring clusters 0 and 3 (M0 and M3 in tables and charts) have more summed fuzzy subsets F4 and F5 (49% in M0 and 38% in M3), while M1 and M6 have more summed F1 and F2 (M1 47%; M6 51%) than the others.

Combining the two datasets (Therapy and Monitoring) in our population, we observed relatively higher frequencies of monitoring cluster M4 in all therapy clusters.

In our population using k-modes clustering the maximum number of therapy clusters per patient ICU stay was 4 (i.e. there were patients who changed therapy pattern and overall duration of therapy to a maximum of 4 different clusters).

Patients with only one therapy cluster during ICU stay had therapy days mostly in cluster T0; patients with 3 clusters in the ICU stay had equal frequencies of the 4 therapy clusters and patients with 4 therapy clusters in ICU stay more frequently had cluster 1 (40%).

We report details about the number of cluster selections in the figures; the same tables for K-means and K-modes clustering results are presented.

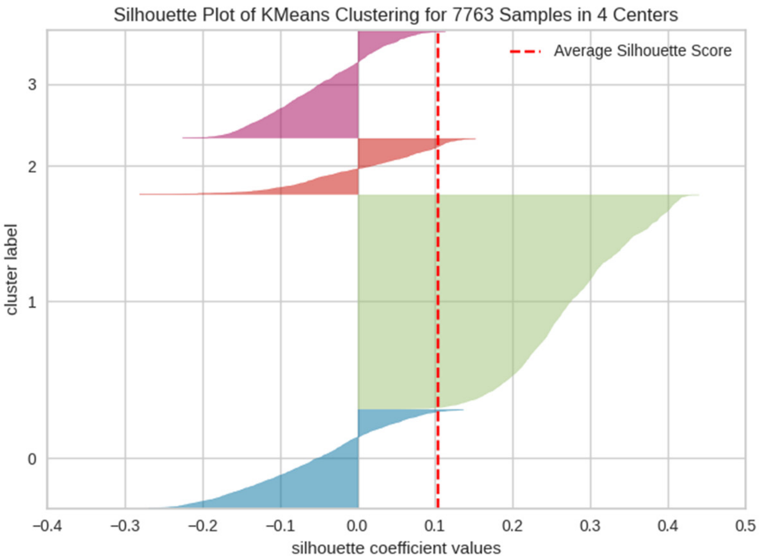

Figure S1. silhouette plot of k-means clustering using 4 centers for antibiotic therapy.

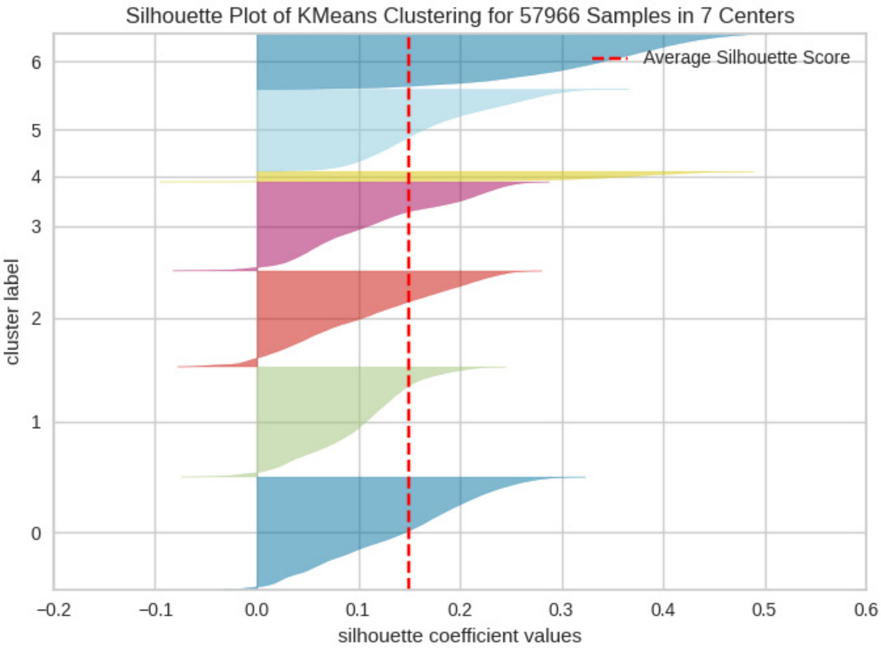

Figure S2. silhouette plot of k-means clustering using 7 centers for monitoring parameters.

Table S1: K-Means clustering. Frequencies of every fuzzy domain subset in each antibiotic therapy cluster.

cluster	N	Non null	F1	F2	F3	F4	F5
**T0**	1626	12861	0.19	0.35	0.21	0.14	0.11
**T1**	3508	17199	0.39	0.50	0.10	0.01	0.00
**T2**	1733	11562	0.21	0.41	0.20	0.12	0.06
**T3**	896	10285	0.13	0.30	0.16	0.15	0.26

(Cluster of therapy: T0, ...T3; Fuzzy categories: F1, ..F5). N: unique antimicrobial day pattern per cluster. Non null: Total of non null fuzzy subsets in the patterns.

Table S2: K-Means clustering. Distribution of frequencies of fuzzy categories (subsets) per monitoring cluster

cluster	N	Non null	F1	F2	F3	F4	F5
**M0**	5747	64563	0.04	0.22	0.39	0.24	0.11
**M1**	10031	75448	0.09	0.27	0.31	0.21	0.12
**M2**	11749	59772	0.07	0.21	0.32	0.26	0.15
**M3**	1083	79445	0.10	0.33	0.29	0.17	0.10
**M4**	9258	57518	0.18	0.37	0.21	0.13	0.10
**M5**	8555	31989	0.07	0.27	0.31	0.21	0.13
**M6**	11543	21367	0.09	0.35	0.31	0.17	0.09

(Monitoring cluster M0, ...M6; Fuzzy categories: F1, … F5). N: unique hourly parameter pattern per cluster. Non null: Total of non null fuzzy subsets in the patterns.

Table S3: K-Means clustering. We show the contingency table between antimicrobial therapy pattern clusters and monitoring pattern clusters.

	M0	M1	M2	M3	M4	M5	M6
**T0**	0.08	0.23	0.18	0.14	0.27	0.09	0.00
**T1**	0.07	0.19	0.21	0.13	0.33	0.07	0.00
**T2**	0.09	0.23	0.21	0.12	0.25	0.09	0.00
**T3**	0.10	0.19	0.23	0.14	0.27	0.07	0.00

(Therapy cluster: T...; Monitoring cluster: M...). Axes X=monitoring cluster; Y=antimicrobial therapy cluster. Chi-square Value 2368.3; $p < 0.05$.

Table S4: K-Means clustering. Basic statistics on ICU stays.

		Nabtcl = 1	Nabtcl = 2	Nabtcl = 3	Nabtcl = 4
**N (clusters)**		3021	1726	648	120
**cluster**	**T0**	0.003	0.19	0.26	0.25
	**T1**	0.993	0.50	0.33	0.25
	**T2**	0.002	0.30	0.21	0.25
	**T3**	0.001	0.01	0.19	0.25

frequencies of Therapy clusters (T...) stratified for number of different therapy clusters during the same ICU stay. Chi-square Value 2845.23; $p < 0.05$.

Table S5: K-Means clustering. Basic statistics on ICU stays

		Nabtcl = 1	Nabtcl = 2	Nabtcl = 3	Nabtcl = 4
**N (hours)**		159212	231714	96682	24295
**cluster**	**M0**	0.07	0.07	0.11	0.09
	**M1**	0.17	0.22	0.28	0.22
	**M2**	0.22	0.19	0.03	0.22
	**M3**	0.12	0.14	0.16	0.15
	**M4**	0.33	0.30	0.33	0.29
	**M5**	0.09	0.07	0.09	0.04
	**M6**	0.00	0.00	0.00	0.00

frequencies of Monitoring clusters stratified for number of different therapy clusters during the same ICU stay. Chi-square Value 22591.2; $p < 0.05$.

Figure3: K-modes Cost plot for Therapy

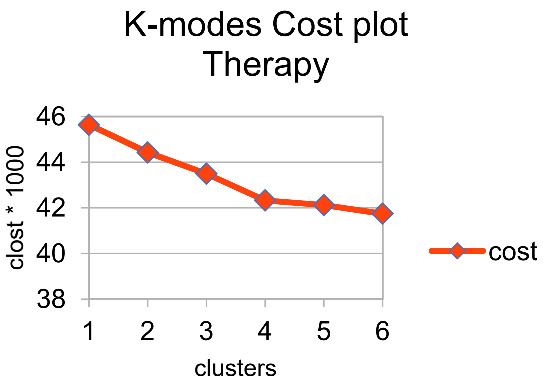

Figure 4: K-modes Cost plot for Monitoring

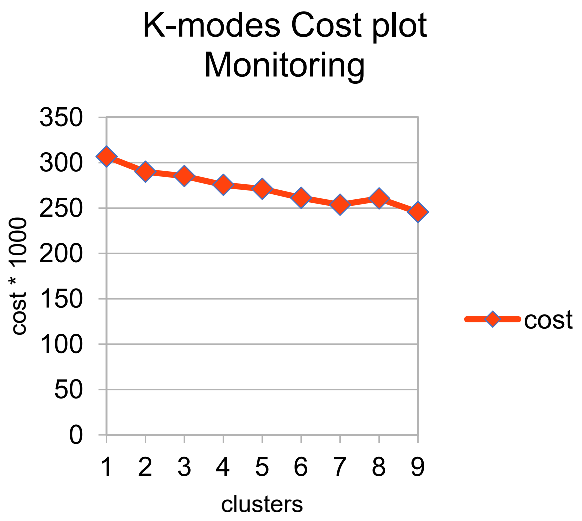

Figure S3 and S4: K-Modes clustering. Cost plot for Monitoring (Figure 3) and Therapy (Figure 4) clustering for number of cluster selection. Cost is the sum of all the dissimilarities between the clusters: we selected the K where an elbow-like bend with a lesser cost value was observed. Values on Y are / 1000 for visualization purposes

Tables 6 and 7 are Tables 1 and 2 in paper numeration. Table 6 shows frequencies of every fuzzy domain subset in each antibiotic therapy cluster. Frequencies of every cluster in each fuzzy domain of hourly monitoring pattern are shown in Table 7.

Table S6: K-Modes clustering. Clustering of unique daily antimicrobial therapy patterns: distribution of frequencies of fuzzy domain subsets per antibiotic therapy cluster.

**cluster**	**N**	**Non null**	**F1**	**F2**	**F3**	**F4**	**F5**
**T0**	3551	10324	0.24	0.38	0.22	0.09	0.07
**T1**	1159	4437	0.11	0.49	0.10	0.11	0.20
**T2**	1569	4257	0.21	0.47	0.13	0.10	0.09
**T3**	1484	5739	0.16	0.34	0.14	0.14	0.22

Clusters of therapy are named from T0 to T3, fuzzy categories from F1 to F5. N: unique antimicrobial day pattern per cluster. Non-null: Total of non null fuzzy subsets in the patterns. Chi-square value 1818.4; p <0.05.

Table S7: K-Modes clustering. Clustering on unique hourly parameter monitoring patterns: distribution of frequencies of sum of fuzzy subsets per monitoring cluster.

**cluster**	**N**	**Non null**	**F1**	**F2**	**F3**	**F4**	**F5**
**M0**	11520	80158	0.07	0.17	0.27	0.34	0.14
**M1**	10017	73525	0.09	0.38	0.34	0.10	0.10
**M2**	14624	93037	0.10	0.27	0.35	0.14	0.14
**M3**	7717	49562	0.08	0.20	0.33	0.30	0.08
**M4**	2391	16732	0.09	0.34	0.28	0.25	0.05
**M5**	3358	22431	0.07	0.32	0.24	0.24	0.13
**M6**	8339	54657	0.12	0.39	027	0.10	0.12

Monitoring clusters are named from M0 to M6, fuzzy categories from F1 to F5. N (unique hourly parameter pattern per cluster). Non-null: Total of non null fuzzy subsets in the patterns. Chi-square value 35848.5; p <0.05.

Table S8: K-Modes clustering.

	M0	M1	M2	M3	M4	M5	M6
**T0**	0.07	0.05	0.16	0.04	0.01	0.01	0.10
**T1**	0.04	0.02	0.07	0.02	0.00	0.01	0.04
**T2**	0.04	0.03	0.07	0.02	0.01	0.01	0.04
**T3**	0.03	0.02	0.05	0.02	0.00	0.01	0.03

Contingency table (frequencies) in the population: X=monitoring cluster (M...); Y=antimicrobial therapy cluster (T…). Chi-square Value 2368.3; *p* <0.05.

Table S9: K-Modes clustering. Basic statistics on ICU stays.

		Nabtcl = 1	Nabtcl = 2	Nabtcl = 3	Nabtcl = 4
**N (clusters)**		2377	1414	306	32
**cluster**	**T0**	0.78	0.38	0.25	0.12
	**T1**	0.00	0.24	0.28	0.40
	**T2**	0.13	0.22	0.24	0.28
	**T3**	0.09	0.16	0.23	0.21

frequencies of Therapy clusters (T...) stratified for number of different therapy clusters (Nabtcl 1 to 4) during the same ICU stay. Chi-square Value 100641,5; *p* <0.05.

Table S10: K-Modes clustering. Basic statistics on ICU stays.

		Nabtcl = 1	Nabtcl = 2	Nabtcl = 3	Nabtcl = 4
**N (hours)**		137667	276361	113197	15965
**cluster**	**M0**	0.16	0.16	0.18	0.23
	**M1**	0.12	0.13	0.12	0.12
	**M2**	0.35	0.34	0.34	0.31
	**M3**	0.09	0.10	0.10	0.09
	**M4**	0.03	0.03	0.04	0.02
	**M5**	0.03	0.03	0.04	0.04
	**M6**	0.22	0.21	0.19	0.19

frequencies of Monitoring clusters stratified for number of different therapy clusters (Nabtcl 1 to 4) during the same ICU stay. Chi-square Value 1918.8; *p* <0.05.

References

1 Papachristou, N.; Barnaghi, P.; Cooper, B.A.; Hu, X.; Maguire, R.; Apostolidis, K.; Armes, J.; Conley, Y.P.; Hammer, M.; Katsaragakis, S.; et al. Congruence Between Latent Class and K-Modes Analyses in the Identification of Oncology Patients with Distinct Symptom Experiences. *J. Pain Symptom Manag.* **2018**, *55*, 318–333.e4. https://doi.org/10.1016/j.jpainsymman.2017.08.020.

2 Rousseeuw, P.J. Silhouettes: A graphical aid to the interpretation and validation of cluster analysis. *J. Comput. Appl. Math.* **1987**, *20*, 53–65.

3 Silhouette Visualizer. Available online: https://www.scikit-yb.org/en/latest/api/cluster/silhouette.html (accessed on 15 January 2022).
